# Occurrence of asymptomatic malaria infection and living conditions in the lowlands of Ethiopia: a community-based cross-sectional study

**DOI:** 10.1186/s40249-022-01018-3

**Published:** 2022-09-05

**Authors:** Endale Mengesha Goshu, Meseret Dessalegne Zerefa, Habteyes Hailu Tola

**Affiliations:** 1 Water and Public Health Stream, Ethiopian Institute of Water Resources, Addis Ababa University, Addis Ababa, Ethiopia; 2 Tuberculosis/HIV Research Directorate, Ethiopian Public Health Institute, P.O. Box 1242, Addis Ababa, Ethiopia

**Keywords:** Occurrence, Asymptomatic, Malaria, Households, Wall, Prevalence, Mosquito, And livestock

## Abstract

**Background:**

A significant decline in malaria burden was documented in previously high burden African countries. Even though the global decline in malaria burden is significant, about 95% of it was typically found in 29 African countries and the decline was affected by COVID-19 in 2020. The considerable reduction in malaria incidence was noted due to effective prevention and treatment efforts, and rapid changes in living conditions. The relationship between the occurrence of asymptomatic malaria infection and household living conditions is well unstudied. This study aimed to determine the association between household living conditions and the occurrence of asymptomatic malaria in the lowlands of Ethiopia.

**Methods:**

A community-based cross-sectional study was conducted from January to March 2021 in twelve villages of Gambella, Southern Nation Nationalities and People Region and Afar in Ethiopia. A total of 1366 households were randomly selected, interviewed, and tested for malaria by rapid diagnostic test and blood film microscopic examination. Multiple logistic regression model was used to determine the independent association between living conditions and asymptomatic malaria infection.

**Results:**

The prevalence of asymptomatic malaria infection among individuals living in dwellings built with traditional floor/wall/roof ranges from 8.1% to 8.4% while it ranges from 2.0% to 4.6% among those living in modern floor/wall/roof houses. Dwellings built with traditional wall materials (*P* = 0.050), spending nights with cattle in the same house (*P* < 0.001), and availability of kitchen in the main house with no partition (*P* = 0.004) were significantly associated with asymptomatic malaria infection.

**Conclusions:**

Asymptomatic malaria infection was 4.3 times higher among occupants residing in dwellings built with traditional wall materials; 5.6 times higher among households spending nights with cattle in the same house, and 2.3 times higher among households with kitchen in the main house with no partition. Therefore, policies and strategies on malaria elimination need to address or target improvements of the above listed living conditions for the community. A multi sectoral action is required to use these social determinants as a vector control strategic addition; and malaria elimination programs are expected to coordinate the implementation.

**Supplementary Information:**

The online version contains supplementary material available at 10.1186/s40249-022-01018-3.

## Background

Till 2019, a significant decline in malaria burden was documented in the previously high burden reporting African countries [1]. However, the estimated malaria cases have increased from 227 million in 2019 to 241 million in 2020, and a 12% increment was noted from 558,000 in 2019 to 627,000 in 2020 in 85 malaria endemic countries [2]. The increment was due to a notable disruption to the declining trend from previous years which was the result of service disruptions from the COVID-19 pandemic [2, 3]. Although there was a significant decline and a slight increment in incidence, about 95% of the global malaria burden was found in 29 African countries and malaria still stands among the top public health priorities for these countries [1]. The reduction in malaria incidence is mainly due to the efforts made in the prevention and treatment of malaria infection and the rapid change in living conditions with socio-economic developments, as individuals spend more on lifestyle changes [4]. Evidence has revealed that improvements in living conditions and housing structure have contributed to malaria prevention [5, 6]. A multi-country survey showed the odds of malaria infection was reduced by 9–14% for modern housing compared with traditional and poorly constructed houses and lower income increased risk of malaria in Sub-Saharan Africa (SSA) [6, 7]. Studies conducted in Korogwe, Tanzania, the southern province of Zambia and Northwest of Burkina Faso showed that the increased housing quality (housing index) decreases the incidence of malaria [8, 9]. A study conducted in Jawi, northwest of Ethiopia, indicated the significant association of the occurrence of malaria infection in a dry season with living in a house with holes in the wall [10]. Data from the Ethiopia malaria indicator survey showed that malaria was found to be higher in thatch and stick/mud roof and earth/local dung plaster floor [11]. Significant reduction of *Anopheles arabiensis* was noted in a randomized trial conducted in south-west Ethiopia on screening of doors, windows, and closing openings on eaves and walls by mud [12, 13].

Mosquito rests in shaded, rough, and cool areas during the daytime and become active at night to get their meal from warm blooded animals and exposure to mosquito bites happen either indoor or outdoor during late hour activities and social gatherings such as religious, cultural, and other activities [14–16]. In a study conducted in rural Indonesia, the occurrence of malaria was 2.8 times higher in individuals who raised their livestock inside their homes compared with those who did not [17]. There are few studies indirectly explained the association between mosquito attracting ability of livestock which increased the probability of malaria infection [18, 19].

A national survey of data on under five children in SSA showed a strong association between drinking unprotected water and an increased risk of malaria infection [20]. The study based on 2007 baseline malaria indicator survey conducted in Southern Nation Nationalities and People Region (SNNPR), Amhara and Oromia regions of Ethiopia showed a higher positive malaria infection among female respondents who used unprotected water [11]. The location of the kitchen could be classified as outside the main house, separate rooms in the main house and in the main house with no separation. Adults and young children were exposed to outdoor biting of mosquitoes as they spent time outside in the kitchen in the Ethiopian highlands [21].

**Fig. 1:**
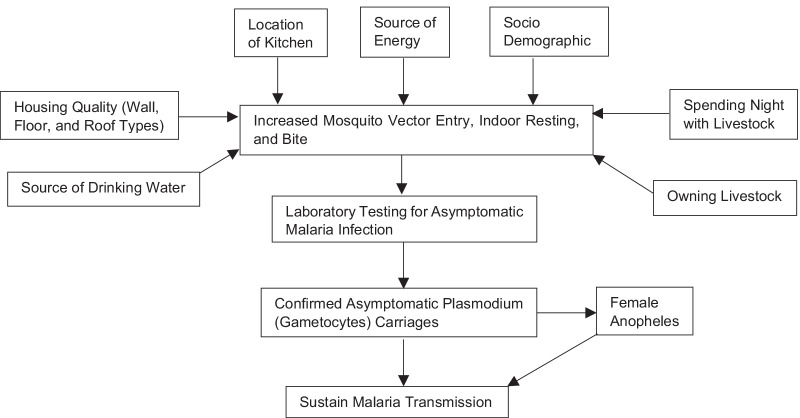
Conceptual framework for the study (developed from literature review)

Most of the studies conducted in Ethiopia focused on the housing conditions and the density of *Anopheles arabiensis* and the relationship between the occurrence of asymptomatic malaria infection and households comprehensive living conditions has not been well studied in Ethiopia. Living conditions such as housing quality (types of walls, roof, floor); location of kitchen (outdoor, indoor with or without separation); energy source; drinking water source; spending night with livestock and socio-demographic variables could increase mosquito vector entry, indoor resting and bite contributing to malaria transmission (Fig. 1). Therefore, this study aimed to determine the association of household living conditions and the occurrence of asymptomatic malaria in the lowlands of Ethiopia.

## Methods

### Study design and areas

A community-based cross-sectional study was conducted in 12 villages of Abobo, Dupti and Abeshge districts of Gambella, Afar and SNNPR national regional states of Ethiopia. Abobo district was found 815 km away from Addis Ababa to the southwest of Ethiopia. The district has a total population of 19,458 and can be explained by a hot and humid climate having nine months of rainy season. Dupti district is 591 km away from Addis Ababa to the north-east of Ethiopia. The district, with a total population of 65,342, shares a similar climatic condition with Abobo [22]. Abeshge district is 167 km away from Addis Ababa to the southwest of Ethiopia. The district is bordered by the Gibe River and has 61,424 inhabitants. The district was bordered by the Gibe river with 61,424 inhabitants [22].

Alwero irrigation dam, Gilgel Gibe power plant dam and Awash irrigation plants were found in Abobo, Abeshge and Dupti districts of Gambella, SNNPR and Afar national regional states respectively [23]. Most human dwellings are built with traditional materials while some of them were built with modern materials. All houses built with a finished wall, roof, and floor materials, are classified as modern houses, while others built with natural or rudimentary materials are classified as traditional houses (Additional file 1: Table S1) [24].

### Sample size and sampling technique

The sample size was calculated by using StatCalc Epi Info version7 software of Centers for Disease Control and Prevention (CDC), Atlanta, Georgia (US). A total of 1390 was calculated using nearby asymptomatic malaria prevalence [25] with 95% confidence level, 3% margin of error and 5% for non-response rate management, and two design effects were considered in the sample size calculation. The households were proportionally allocated to each selected village based on their respective population size.

**Fig. 2:**
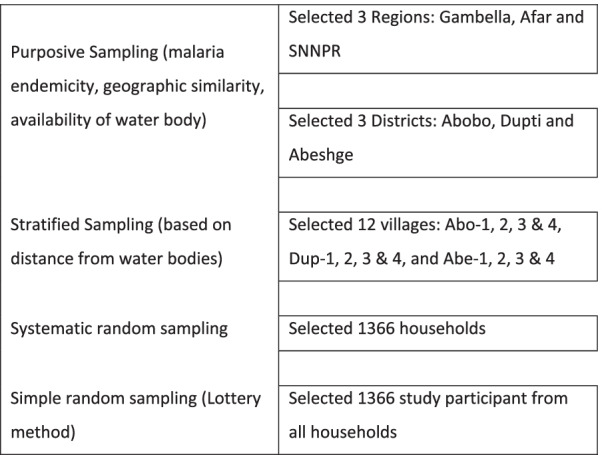
Sampling techniques

Regions and districts were selected purposely based on the epidemiological distribution of malaria, availability of water bodies and geographic similarities. The villages in each district were stratified into four strata based on distance from water bodies. Each stratus was selected as a cluster within ≤ 3 km, 4–6 km, 7–9 km and > 9 km. Households in each stratus were selected by systematic random sampling method from a health post household registration book. Study participants were selected by a simple random sampling technique (lottery method) from each household. January and February months were non-rainy seasons and selected for dry season data collection [26] (Fig. 2).

### Data collection tools and procedures

A total of 1500 tool kits, consisting of one rapid diagnostic test (RDT) kit, two microscope slides and one structured questionnaire, were distributed to data collectors in each region. Enrollment was initiated after introduction to the main objective of the study and once written consent was obtained from eligible participants. Data collection was carried out using a structured questionnaire. Blood sample collection for microscopy and RDT testing was conducted in the field while microscopic examination of slides was performed in selected health facility laboratories. During data collection, clinical malaria cases who were excluded from this study were managed as per the national guidelines at health post level and nearby health center level. One supervisor was assigned per region for two teams and visited their respective teams twice a day for data quality assurance, malaria RDT testing and blood film preparation procedures. The filled questionnaires were checked randomly for completeness and consistency. All the filled and verified questionnaires with prepared blood film microscope slides were collected, sealed, and transported by the supervisors to the selected health centers. The initial microscope slide examination was performed at health center and hospital level and examined slides were transported to regional laboratories for quality assurance as per the national recommendation.

### Data management and analysis

The collected data was double entered into the spread sheets to minimize mismatch and out of range errors. The entered data was analyzed by IBM statistical package for social sciences (SPSS) for Windows, Version 20.0. General characteristics of the data and statistical distribution of households’ living conditions were examined by descriptive analysis. The association between occurrence of asymptomatic malaria and possible living conditions was explored by using the Chi squared test. Living conditions such as housing quality, owning, and spending the night with livestock, source of drinking water and other variables were tested for association with the outcome variable (Table 1). The association was considered significant at *P*-value ≤ 0.05. Variables scored the *P*-value of less or equal to 0.2 during bi variate analysis were included to multivariate logistic regression.

**Table 1:** Frequency, and association of asymptomatic malaria prevalence and living condition

Variable	Malaria lab result		U*OR* (95% *CI*)	*P*–value
	Negative *n* (%)	Positive *n* (%)		
Residence				
Urban	389 (95.3)	19 (4.7)	1	
Rural	872 (91.0)	86 (9.0)	2.0 (1.2–3.4)	0.007
Age, years				
< 5	68 (88.3)	9 (11.7)	1	
5–14	380 (92.2)	32 (7.8)	0.6 (0.3–1.4)	0.258
15–44	647 (93.0)	49 (7.0)	0.6 (0.3–1.2)	0.146
45–64	104 (91.2)	10 (8.8)	0.7 (0.3–1.9)	0.510
> 64	62 (91.6)	5 (7.5)	0.6 (0.2–1.9)	0.397
Sex				
Female	686 (93.0)	52 (7.0)	1	
Male	575 (91.6)	53 (8.4)	1.2 (0.8–1.8)	0.336
Educational status				
High school and above	257 (92.4)	21 (7.6)	1	
Elementary	494 (92.7)	39 (7.3)	1.0 (0.6–1.7)	0.903
Illiterate	510 (91.9)	45 (8.1)	1.1(0.6–1.9)	0.780
Occupational status				
Government employee	74 (91.4)	7 (8.6)	1	
Self-employee	190 (93.6)	13 (6.4)	0.7 (0.3–1.9)	0.507
Student	429 (92.3)	36 (7.7)	0.9 (0.4–2.1)	0.781
Housewife	362 (94.0)	23 9(6.0)	0.7 (0.3–1.6)	0.377
Farmer	206 (88.8)	26 (11.2)	1.3 (0.6–3.2)	0.519
Family size				
Less or equal two 2	421 (93.1)	31 (6.9)	1	
Above 2	840 (91.9)	74 (8.1)	1.2 (0.8–1.8)	0.420
Availability of electronic device				
Yes	329 (94.0)	21 (6.0)	1	
No	932 (91.7)	84 (8.3)	1.4 (0.9–2.3)	0.171
Location of kitchen				
Outside the house	459 (94.8)	25 (5.2)	1	
Separate room in the main house	126 (93.3)	9 (6.7)	1.3 (0.6–2.9)	0.500
In the main house with no separation	676 (90.5)	71 (9.5)	1.9 (1.2–3.1)	0.006
Source of energy for cooking				
Electricity	31 (91.2)	3 (8.8)	1	
Charcoal	265 (96.7)	9 (3.3)	0.4 (0.1–1.4)	0.131
Wood	965 (91.2)	93 (8.8)	1.0 (0.3–3.3	0.995
Owning of livestock, herds, or farm animals				
No	492 (92.3)	41 (7.7)	1	
Yes	769 (92.3)	64 (7.7)	1.0 (0.7–1.5)	0.995
Spending night with cattle in the same house				
No	1022 (94.5)	60 (5.5)	1	
Yes	239 (84.2)	45 (15.8)	3.2 (2.1–4.8)	0.000
Household floor type				
Modern	88 (97.8)	2 (2.2)	1	
Traditional	1173 (91.9)	103 (8.1)	0.3 (0.1–1.1)	0.061
Household wall type				
Modern	145 (98.0)	3 (2.0)	1	
Traditional	1116 (91.6)	102 (8.4)	4.4 (1.4–14.1)	0.012
Household roof type				
Modern	165 (95.4)	8 (4.6)	1	
Traditional	1096 (91.9)	97 (8.1)	0.5 (0.3–1.1)	0.111
Source of drinking water				
Improved source of drinking water	277 (94.9)	15 (5.1)	1	
Other Improved (public tap) source drinking water	550 (90.3)	59 (9.7)	2.0 (1.1–3.6)	0.022
Un improved source of drinking water	434 (93.3)	31 (6.7)	1.3 (0.7–2.5)	0.392

*UOR*: Unadjusted odd ratio; *CI*: Confidence interval

## Results

### Socio-demographic characteristics of study participants

Among the total participants, 51% (696) of them were in the age range of 15–44 years and 54% (738) of them were female. In relation to the educational status of the study participants, 555 (40.6%), 533 (39.0%) and 278 (20.4%) were illiterate, elementary, and high school complete, respectively. Of the total households, 801(58.6%) of them treat their drinking water while 833 (61%) own livestock and 1082 (79.2%) spend their night in the same house (Table 1).

### Housing condition

This study enrolled 1366 households and only one eligible member of each household was interviewed and tested for asymptomatic malaria parasite. About 70% of the houses were in the rural areas. Among the total houses visited, 1276 (93.4%), 1218 (89.2%) and 1193 (87.3%) of them were constructed with traditional floors, walls, and roofs respectively. About 747 (54.7%) of houses have no separation of the main house from their kitchen, while others do or are placed outside. Only 34 (2.5%) houses have access to electricity as a source of energy for cooking, while 1058 (77.5%) of them use wood and the rest charcoal. Of the total 1366 households, 465 (34%) were obtained water supply from unimproved source, while 609 (44.6%) from improved (public tap) and 292 (21.4%) improved (piped) type water sources (Table 1).

### Occurrence of asymptomatic malaria infection in the study sites

The prevalence of asymptomatic *Plasmodium* carriage (*P. falciparum*, *P. vivax* and mixed species) was 8.1% (111/1366) as determined by microscopy, while the prevalence as determined using RDT was 9.3% (127/1366).

### Association of asymptomatic malaria infection and living conditions

The occurrence of asymptomatic malaria infection was strongly associated with kitchen being in the main house with no separation (unadjusted odds ratio (U*OR*) = 1.9; 95% confidence interval (*CI*): 1.2–3.1; *P* = 0.006), individuals spending nights with cattle in the same house (U*OR* = 3.2; 95% *CI* (2.1–4.8); *P* = 0.000), houses built with traditional wall [U*OR* = 4.4; 95% *CI* (1.4–14.1); *P* = 0.012], houses supplied with other Improved (public tap) source of drinking water (U*OR* = 1.981; 95% *CI* : 1.1–3.6; *P* = 0.022), and being a rural house (U*OR* = 2.0; 95% *CI*: 1.2–3.4; *P* = 0.007) (Table 1).

### Independent factors analysis

In logistic regression model analysis, the walls of the dwellings built with traditional materials [adjusted odds ratio (A*OR*) = 4.3; 95% *CI*: 1.0–18.4; *P* = 0.047], spending nights with cattle in the same house (A*OR* = 5.6; 95% *CI* 3.5–9.1; *P* = 0.000), and availability of kitchen in the main house with no separation (A*OR* = 2.3; 95% *CI*: 1.3–4.2; *P* = 0.005), were significantly associated with asymptomatic malaria infection after adjusting for the effect of other confounding variables (Table 2). However, houses supplied with public tap water sources for drinking and residence lost their significance after adjusting for the confounder.

**Table 2:** Independent effect of living conditions on the prevalence of asymptomatic malaria

Variable	Malaria lab result		A*OR* (95% *CI*)	*P-*value
	Negative *n* (%)	Positive *n* (%)		
Residence				
Urban	389 (95.3)	19 (4.7)	1	
Rural	872 (91.0)	86 (9.0)	1.8 (0.6–5.4)	0.289
Age, years				
< 5	68 (88.3)	9 (11.7)	1	
6–14	380 (92.2)	32 (7.8)	0.7 (0.3–1.6)	0.418
15–44	647 (93.0)	49 (7.0)	0.7 (0.3–1.6)	0.437
45–64	104 (91.2)	10 (8.8)	0.9 (0.3–2.5)	0.818
> 64	62 (91.6)	5 (7.5)	0.7 (0.2–2.2)	0.498
Availability of electronic device				
Yes	329 (94.0)	21 (6.0)	1	
No	932 (91.7)	84 (8.3)	1.1 (0.6–1.8)	0.718
Location of kitchen				
Outside the house	459 (94.8)	25 (5.2)	1	
Separate room in the main house	126 (93.3)	9 (6.7)	1.4 (0.6–3.5)	0.398
In the main house with no separation	676 (90.5)	71 (9.5)	2.3 (1.3–4.2)	0.005
Source of energy for cooking				
Electricity	31 (91.2)	3 (8.8)	1	
Charcoal	265 (96.7)	9 (3.3)	0.5 (0.1–2.3)	0.388
Wood	965 (91.2)	93 (8.8)	1.2 (0.3–4.4)	0.838
Spending night with cattle in the same house				
No	1022 (94.5)	60 (5.5)	1	
Yes	239 (84.2)	45 (15.8)	5.6 (3.5–9.1)	0.000
Household floor type				
Modern	88 (97.8)	2 (2.2)	1	
Traditional	1173 (91.9)	103 (8.1)	1.0 (0.2–6.4)	0.862
Household wall type				
Modern	145 (98.0)	3 (2.0)	1	
Traditional	1116 (91.6)	102 (8.4)	4.3 (1.0–18.4)	0.047
Household roof type				
Modern	165 (95.4)	8 (4.6)	1	
Traditional	1096 (91.9)	97 (8.1)	0.7 (0.3–1.7)	0.422
Source of drinking water				
Improved source of drinking water	277 (94.9)	15 (5.1)	1	
Other Improved (public tap) source drinking water	550 (90.3)	59 (9.7)	0.7 (0.2–2.1)	0.533
Un improved source of drinking water	434 (93.3)	31 (6.7)	0.4 (0.1–1.0)	0.059

*AOR*: Adjusted odd ratio; *CI*: Confidence interval

## Discussion

The key determinants for epidemiology of vector-borne disease are the variation in vulnerability level of the specific population. Living conditions are the key factors that determine the malaria vulnerability of a given population. In the current study, characteristics of living conditions such as housing quality (types of walls, roof, floor); location of kitchen (outdoor, indoor with or without separation); energy source; drinking water source; spending night with livestock and socio-demographic variables were tested. The argument is whether above indicated living conditions could increase the probability of asymptomatic malaria infection through increasing mosquito vector entry, indoor resting, bite and contribute for malaria transmission. This research findings revealed that living conditions such as wall type of the house, spending night with livestock in the same house and the location of kitchen were significantly associated with prevalence of asymptomatic malaria infection.

As per the findings of this research, the association between asymptomatic malaria prevalence and housing structure showed that better housing was associated with reduced odds of asymptomatic malaria and it’s an effective addition to vector control strategies. The prevalence of asymptomatic malaria infection among occupants living in houses built with traditional floor/wall/roof ranges from 8.1% to 8.4% while it ranges from 2.0% to 4.6% among those living in modern floor/wall/roof houses. It was 4.3 times higher in occupants residing in a house built with traditional wall materials (natural or rudimentary) compared with occupants residing in a house built with modern (finished- cement or brick) wall materials.

Previous findings on the association between malaria infection and housing quality indicated the prevalence of malaria among occupants residing in houses built in traditional materials was 8.8% while it ranges from 1.4 to 1.6% among occupants living in houses built with modern materials, supported the result of the current study [27, 28]. Significant association between housing quality and asymptomatic malaria infection found in this study was supported by studies conducted in Southern Zambia, Uganda, Swaziland, Northern Botswana, and a working paper series on quality of homes in Africa [16, 27–30].

In the current study, spending nights with cattle in the same house was significantly associated with increased asymptomatic malaria infection. The occurrence of asymptomatic malaria infection was 5.6 times higher in individuals who spent their nights with their cattle compared with those who did not. This may be due to the attractive nature of livestock for zoophilic and opportunistic mosquito vectors to households and once they get into the households, the opportunistic mosquito prefers to bite human skin as it is easy for their proboscis. Previous study findings conducted in southern Ethiopia and Indonesia on the association of malaria infection and keeping medium sized animals in the same house with family members supported the findings of this study and showed a 3 times higher risk of contracting malaria infection [17, 19].

The location of the kitchen was classified as outside the main house, separate rooms in the main house and in the main house with no separation and analyzed for association with asymptomatic malaria infection. In logistic regression model analysis, the odds of kitchen location in the main house with no separation was more than two times compared with households who kept their kitchen outside the main house. This association may be due to the kitchen location serving as proxies for indoor resting of the households during non-smoky hours. This finding was supported by a study conducted among children in the Ethiopian highlands [21].

### Strength and limitation of the study

The strength of the study includes generalizability to similar lowland areas in the continent Africa and representativeness of study area (selection of districts from three different regions which have similar characteristics (altitude, weather condition, water bodies and malaria burden). In addition to its large sample size, systematic and simple random sampling techniques were used for household (household registration logbook) and study subject selection respectively. Adjustments for confounders also applied during data analysis.

Though this is one of a few studies that tried to determine the association between asymptomatic malaria infection and comprehensive living conditions, it should be interpreted cautiously as there are some limitations. For instance, it applied observational studies, limiting causality implications between asymptomatic malaria carriages, and living conditions. In addition, we couldn’t directly show the association of asymptomatic malaria infection and number holes, cracks, open doorways, or eaves, as we didn’t collected information. We recommend future studies to include the association of number of holes and crack with prevalence of asymptomatic malaria.

## Conclusions

The findings of current study supported, improvements in housing quality, spending nights away from livestock and owning of kitchen separated from main living house as a worthwhile consideration in malaria elimination efforts as additional value over existing vector control packages. The ministry of health national malaria elimination programs needs to include the above indicated key factors in their vector control strategies through coordination of all stakeholders as it requires a multi sectoral efforts. The coordination needs to include ministry of agriculture for livestock management; ministry of urban development, housing and construction for improved quality and low-cost housing design and advocacy; and ministry of Education for intensified health education at school level for malaria endemic areas. It is also recommended that application of existing malaria vector control and personal protection methods and surveillance need to be focused and intensive for the poorer sections of the population with greater risk of malaria.

## Supplementary Information

**Additional file 1: S1.** Operational definition

## Data Availability

The datasets used and/or analyzed during the current study are available with principal investigator.

## Abbreviations

A*OR*: Adjusted odds ratio
*CI*: Confidence interval
EIWR: Ethiopian Institute of Water Resources
IRB: Institutional Review Board
Km: Kilometer
n: Number
RDT: Rapid diagnostic test
SNNPR: Southern Nation Nationality People Region
SPSS: Statistical package for social sciences
UOR: Unadjusted odds ratio
SSA: Sub-Saharan Africa
