# Supplementary material for: Occurrence of asymptomatic malaria infection and living conditions in the lowlands of Ethiopia: a community-based cross-sectional study
Source: Infect Dis Poverty. 2022 Sep 5;11:94. doi: 10.1186/s40249-022-01018-3 (PMC9444277; doi:10.1186/s40249-022-01018-3)
Supplement: Supplementary file 1 — Additional file 1: S1. Operational definition [file 40249_2022_1018_MOESM1_ESM.docx]

## **Operational definition**

Table 1: Operational definitions of key variables and concepts

| **Variable** | **Definition** |
| --- | --- |
| Traditional house | Houses built by natural or rudimentary wall, roof, and floor materials |
| Modern house | Houses built by finished wall, roof, and floor materials |
| Finished wall materials | Material used to build the walls of the dwellings that include bricks, cement blocks, cement, stone with lime or cement, wood planks or shingles, and covered adobe |
| Natural or rudimentary wall materials | Material used to build the walls of the dwellings that include reused wood planks, cane, palm, or stone with mud, uncovered adobe, plywood, cardboard, and dirt, bamboo. |
| Finished roof materials | Material used to build the roofs of the dwellings that include calamine or cement fiber, metal, wood, and roofing shingles, ceramic tiles, cement |
| Natural or rudimentary roof materials | Material used to build the roofs of the dwellings that include rustic matting, bamboo, thatch, palm leaf, sod, wood planks, and cardboard. |
| Finished floor materials | Material used to build the floor of the dwellings that include vinyl or asphalt strips, ceramic tiles, cement, and carpet, parquet, or polished wood |
| Natural or rudimentary floor materials | Material used to build the floor of the dwellings that include sand, dung, earth, wood planks, bamboo, and palms. |
| Improved source of drinking water | Piped household water connection located inside the user’s dwelling, plot or yard or public taps or standpipes, tube wells or boreholes, protected dug wells, protected springs, and rainwater collection. |
| Other improved sources of drinking water | Source of drinking water that include protected springs, rainwater collection, tube wells or boreholes, public taps, or standpipes and, protected dug wells, |
| Unimproved source of drinking water | Source of drinking water that include unprotected dug well, unprotected spring, cart with small tank/drum, tanker truck, and surface water (river, dam, lake, pond, stream, canal, irrigation channels), bottled water (21). |
